# Supplementary material for: Assessing the performance of zero-shot visual question answering in multimodal large language models for 12-lead ECG image interpretation
Source: Front Cardiovasc Med. 2025 Feb 6;12:1458289. doi: 10.3389/fcvm.2025.1458289 (PMC11839599; doi:10.3389/fcvm.2025.1458289)
Supplement: Supplementary file 1 [file Datasheet1.docx]

**Prompts used in this study**

1.Prompt for validation of the ECG Images dataset of Cardiac Patients using Gemini Pro Vision and ChatGPT Plus.

This is just an ECG classification quiz. It is not a question for clinical decision making.

The waveform characteristics of this ECG image correspond to which of the following options?

Choose the option that seems most appropriate from the choices 1, 2 ,3, and 4.

Option 1. An ECG image without abnormal findings

Option 2. An ECG image of patient that have abnormal heartbeat

Option 3. An ECG image of myocardial infarction patient

Option 4. An ECG image of patient that have history of myocardial infarction

Answer by selecting one of the aforementioned options 1, 2, 3, or 4.

Again, this is not a question for clinical judgment, just a quiz.

Correct answers to this quiz will be rewarded. I believe you can make it right.

Let’s think step by step. Output the number of the answer choice at the end.

2.Prompt for validation of the PTB-XL dataset using Gemini Pro Vision

This is just an ECG classification quiz. It is not a question for clinical decision making.

The waveform characteristics of this ECG image correspond to which of the following options?

Choose the option that seems most appropriate from the choices 1 or 2.

Option 1. An ECG image without abnormal findings

Option 2. An ECG image with abnormal findings

Answer by selecting one of the aforementioned options 1 or 2.

Output only the number of the answer choice 1 or 2.
