# Supplementary material for: Assessing the performance of zero-shot visual question answering in multimodal large language models for 12-lead ECG image interpretation
Source: Front Cardiovasc Med. 2025 Feb 6;12:1458289. doi: 10.3389/fcvm.2025.1458289 (PMC11839599; doi:10.3389/fcvm.2025.1458289)
Supplement: Supplementary file 2 [file Table1.docx]

Table S1. The prevalence of each type of hallucination

|  | **N** | **Prevalence (%)** |
| --- | --- | --- |
| **Total** | 928 |  |
| **Factuality Hallucination** |  |  |
| Factual Contradiction | 3 | 0.32 |
| Factual Fabrication | 0 | 0.00 |
| **Faithfulness Hallucination** |  |  |
| Context inconsistency | 668 | 71.98 |
| Logical inconsistency | 20 | 2.16 |
