# Supplementary material for: Assessing the performance of zero-shot visual question answering in multimodal large language models for 12-lead ECG image interpretation
Source: Front Cardiovasc Med. 2025 Feb 6;12:1458289. doi: 10.3389/fcvm.2025.1458289 (PMC11839599; doi:10.3389/fcvm.2025.1458289)
Supplement: Supplementary file 3 [file Table2.docx]

Table S2. Cohen's Kappa coefficients between evaluators

| **Evaluation items** | | **Cohen's kappa coefficient (95% CI)** |
| --- | --- | --- |
| **Medical assumptions** | Does it include a description of medical prerequisites? | 0.937 (0.902 - 0.965) |
| **Image findings** | Does it include a description of ECG findings? | 0.969 (0.888 - 1.000) |
|  | Is there any abnormal finding not described? | 0.998 (0.993 - 1.000) |
|  | Is there any abnormal finding described as a different abnormality? | 0.963 (0.923 - 0.992) |
|  | Is there any abnormal finding correctly identified as abnormal? | 0.987 (0.966 - 1.000) |
|  | Is there any normal finding incorrectly labeled as abnormal? | 0.997 (0.990 - 1.000) |
|  | Is there any normal finding correctly identified as normal? | 0.996 (0.989 - 1.000) |
| **Logical consistency** | Does a contradiction exist in the logic of answer selection? | 0.974 (0.907 - 1.000) |

CI, Confidence interval
