# Supplementary figures and images for: Assessing the performance of zero-shot visual question answering in multimodal large language models for 12-lead ECG image interpretation
Source: Front Cardiovasc Med. 2025 Feb 6;12:1458289. doi: 10.3389/fcvm.2025.1458289 (PMC11839599; doi:10.3389/fcvm.2025.1458289)

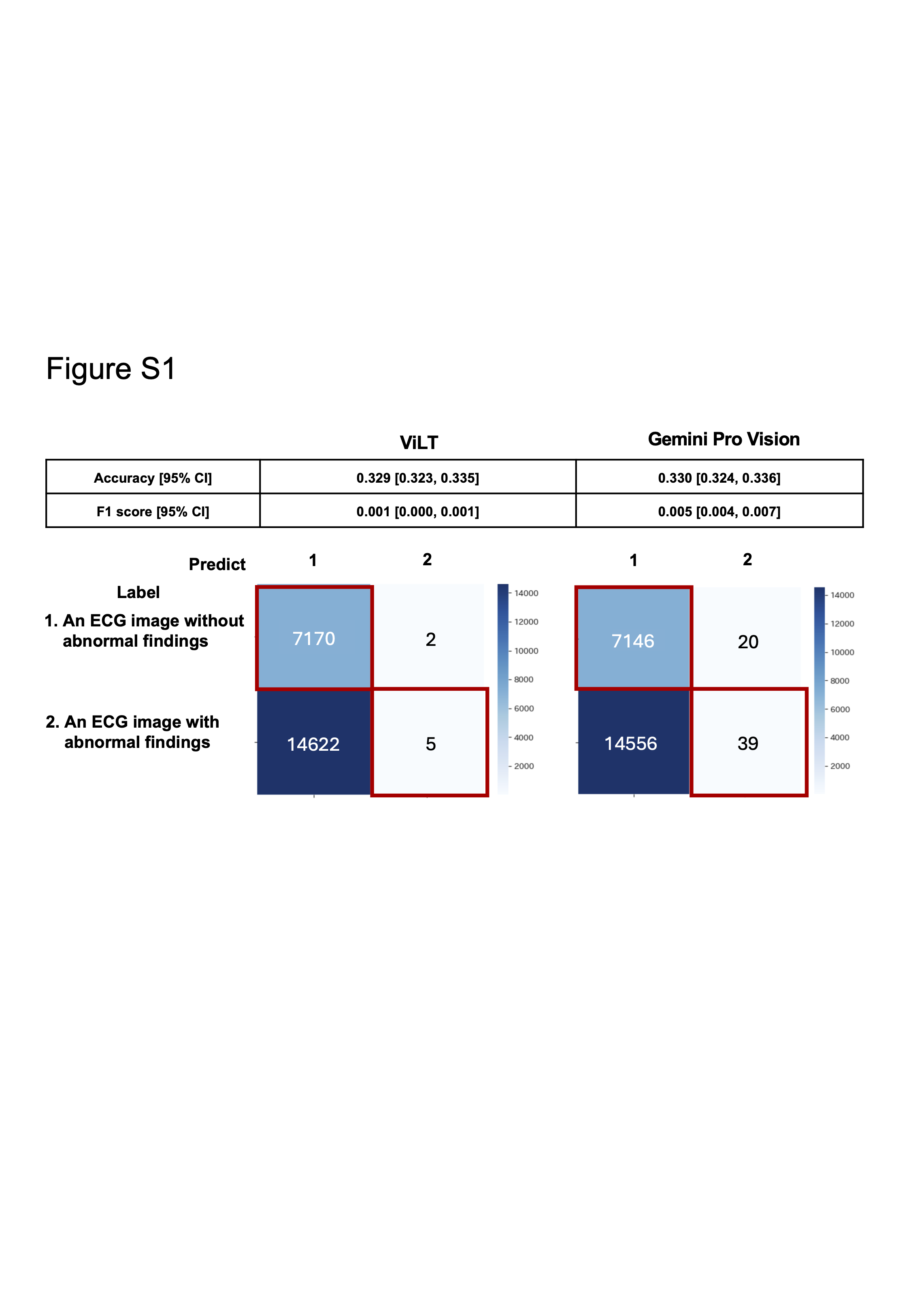

Supplement: Supplementary Figure S1 — Prediction results and confusion matrix for classification of 12-lead ECG images on PTB-XL dataset. Performance indices for each model are displayed at the top of the figure, and the confusion matrix is displayed at the bottom of the figure. Red squares in the confusion matrix indicate correct cases. [file Image1.tiff]
